# Fabrication of MNPs/rGO/PMMA Composite for the Removal of Hazardous Cr(VI) from Tannery Wastewater through Batch and Continuous Mode Adsorption

**DOI:** 10.3390/ma14226923

**Published:** 2021-11-16

**Authors:** Rahman Ullah, Waqas Ahmad, Muhammad Yaseen, Mansoor Khan, Mehmood Iqbal Khattak, Badrul Mohamed Jan, Rabia Ikram, George Kenanakis

**Affiliations:** 1 Institute of Chemical Sciences, University of Peshawar, Peshawar 25120, Khyber Pakhtunkhwa, Pakistan; rahmandawar@uop.edu.pk (R.U.); myyousafzai@gmail.com (M.Y.); 2 Department of Chemistry, Kohat University of Science and Technology, Kohat 26000, Khyber Pakhtunkhwa, Pakistan; mansoor009988@gmail.com; 3 Material Science Center (PCSIR) Laboratories Complex, Peshawar 25120, Khyber Pakhtunkhwa, Pakistan; mahmood2162002@gmail.com; 4 Department of Chemical Engineering, University of Malaya, Kuala Lumpur 50603, Malaysia; 5 Institute of Electronic Structure and Laser, Foundation for Research and Technology-Hellas, N. Plastira 100, Vasilika Vouton, GR-70013 Heraklion, Crete, Greece; gkenanak@iesl.forth.gr

**Keywords:** reduced graphene oxide, MNPs/rGO/PMMA composite, hexavalent chromium, tannery wastewater, fixed bed adsorption

## Abstract

Herein, we report the synthesis of magnetic nanoparticle (MNP)-reduced graphene oxide (rGO) and polymethylmethacrylate (PMMA) composite (MNPs/rGO/PMMA) as adsorbent via an in situ fabrication strategy and, in turn, the application for adsorptive removal and recovery of Cr(VI) from tannery wastewater. The composite material was characterized via XRD, FTIR and SEM analyses. Under batch mode experiments, the composite achieved maximum adsorption of the Cr(VI) ion (99.53 ± 1.4%, i.e., 1636.49 mg of Cr(VI)/150 mg of adsorbent) at pH 2, adsorbent dose of 150 mg/10 mL of solution and 30 min of contact time. The adsorption process was endothermic, feasible and spontaneous and followed a pseudo-2nd order kinetic model. The Cr ions were completely desorbed (99.32 ± 2%) from the composite using 30 mL of NaOH solution (2M); hence, the composite exhibited high efficiency for five consecutive cycles without prominent loss in activity. The adsorbent was washed with distilled water and diluted HCl (0.1M), then dried under vacuum at 60 °C for reuse. The XRD analysis confirmed the synthesis and incorporation of magnetic iron oxide at 2θ of 30.38°, 35.5°, 43.22° and 57.36°, respectively, and graphene oxide (GO) at 25.5°. The FTIR analysids revealed that the composite retained the configurations of the individual components, whereas the SEM analysis indicated that the magnetic Fe_3_O_4_–NPs (MNPs) dispersed on the surface of the PMMA/rGO sheets. To anticipate the behavior of breakthrough, the Thomas and Yoon–Nelson models were applied to fixed-bed column data, which indicated good agreement with the experimental data. This study evaluates useful reference information for designing a cost-effective and easy-to-use adsorbent for the efficient removal of Cr(VI) from wastewater. Therefore, it can be envisioned as an alternative approach for a variety of unexplored industrial-level operations.

## 1. Introduction

Over the years, enormous studies have been investigated for wastewater treatments with the breakthrough of research using value-added nanomaterials, including NPs and nanocomposites [1,2]. Tanning is one of the global water-and-soil polluting industries due to its high environmental risks [3]. The usage of chemicals during the tanning process produces a large amount of carcinogenic mobilization of toxic metals, which is the vital concern of the current era [4]. Chrome tanning relies on chromium salts and liquors to minimize hazardous environmental impacts. On the other hand, vegetable tanning is a natural process of tree tannins and water [5]. Among these common tanning procedures, chrome tanning is highly favorable due to its low cost and high productivity [6].

There is a continuous shift of production wastewater, which is highly polluted, containing both inorganic and organic pollutants from the tanneries [7]. Notably, the presence of organic pollutants comprises blood particles, flesh, soluble proteins and hair [8]. Conversely, the inorganic pollutants used as tanning agents are typically salts of various metals, such as Chromium (Cr-III) sulphate and chrome alum [9]. Cr, in aqueous solution, exists in two stable oxidation states (Cr-III and Cr-VI), where both have diverse biological, chemical and environmental possessions [10]. Cr(III) is a moderately insoluble and useful micronutrient for animals, plants and humans. As compared to Cr(III), Cr(VI) is 100 times more contaminant and hazardous and 1000 times more mutagenic and carcinogenic for living organisms due to its oxidizing ability [11]. The occurrence of Cr(VI) in water beyond permissible limits causes liver, cancer and skin disturbance, bringing about ulcer development, diarrhea, hemorrhage, vomiting and damage to the kidneys [12]. According to the World Health Organization (WHO), the acceptable amount of Cr in drinking water is 0.1 mg·L^−1^, while, in industrial wastewater, it is 0.05 mg·L^−1^ and 5 mg·L^−1^ for Cr(VI) and Cr(III), respectively [13]. Therefore, it is a substantial task to discover facile and cost-effective ways to eliminate Cr(VI) from environmental water.

Various techniques have been applied for the effective and economical elimination of Cr(VI) from industrial effluents, including electrochemical technologies [14], use of chemical coagulation solvents [15], extraction [16], ion exchange [17], membrane filtration [18] and adsorption [19]. The remarkable advantages and disadvantages of these techniques have been critically reviewed [5,11]. It has been observed that electrochemical techniques represent low cost and high selectivity along with the fruitless recovery of treated metals for a recycled, high volume of sludge formation [9,14]. Likewise, the ion exchange and membrane filtration processes are restricted due to their selectivity, high operational cost and maintenance issues [20]. However, the adsorption process, by all accounts, has shown unrivaled outcomes due to its minimal effort, high effectiveness and selectivity [21].

Among the nanomaterials, GO has gained enormous attention due to its excellence in thermal, electronic and mechanical impendence [22]. Owing to its high surface area and tremendous adsorption capacity, GO has been utilized as an exceptional adsorbent for the adsorption of heavy metals [23,24]. Very recent studies have utilized novel GO–ionic liquid composites [25], GO/chitosan [26] and Fe_2_O_3_-GO-chitosan composites [27], for enhanced Cr(VI) adsorption.

Besides, MNPs have been employed for the adsorption of substantial metals from wastewater [28] MNPs demonstrate exceptional paramagnetic properties and have a rich surface, which makes their target to be easily separated from the aqueous medium using external magnetic field [29]. However, the adsorption properties of MNPs are limited due to their mobility and aggregation in aqueous media [30]. The MNPs may be combined into composites with other active adsorbents, such as GO, to eliminate the operations of filtration, centrifugation and aggregation and, eventually, to increase the adsorption capacity [23,31]. A variety of magnetized adsorbents has proved high adsorption efficiency using different types of pollutants, including Cr from water [30,32]. The incorporation of a polymer matrix to these nanomaterials may also help reducing their aggregation and enhance their adsorption capacity by offering the addition of a reactive functional group as adsorption site. The chemical and mechanical stability of the adsorbent may also be enhanced with the incorporation of a polymer matrix [33]. 

In this study, we fabricated a novel composite of MNPs with reduced GO (rGO) and polymethylmethacrylate (MNPs/rGO/PMMA) through in situ polymerization and applied it for the adsorptive removal and recovery of Cr(VI) from tannery wastewater. Polymethylmethacrylate (PMMA) has high performance in multipurpose products due to its outstanding mechanical and physical properties. It is resistant to oils, waxes, greases, acids, bases, ozone, weathering and water immersion, which can withstand high temperatures (160 °C). Additionally, PMMA provides multiple carboxylate functionalities for binding the Cr ions, which impart the dispersion properties to the composite and inhibit particle aggregations of rGO and MNPs. Therefore, PMMA was selected as the polymer matrix for the preparation of a composite material in this work. rGO was magnetized using FeCl_3_ and FeSO_4_ salts, using an in situ magnetization process. The synthesized composite was characterized using the scanning electron microscopy (SEM), Fourier transform infra-red spectroscopy (FTIR) and X-rays diffraction (XRD) techniques. The adsorption performance of MNPs/rGO/PMMA for Cr(VI) was evaluated using batch adsorption techniques and fixed-bed column-based experimental set up from model aqueous solution and tannery wastewater.

## 2. Experimental Procedure

### 2.1. List of Chemicals and Standards

In this study, all the chemicals used were of analytical grade and were used without further purification. The MMA monomer and 2,2-Azobisisobutyronitrile (AIBN) were provided by Daejung chemicals, (Siheung-si, Korea). Acetone (CH_3_COCH_3_), sulfuric acid (H_2_SO_4_, 98%), hydrogen peroxide (H_2_O_2_, 30%), sodium nitrate (NaNO_3_, 99%) and hydrazine hydrate were purchased from Merk (Darmstadt, Germany). Ferric chloride (FeCl_3_, 97%) and ferrous sulfate (FeSO_4_.6H_2_O, 97%) were provided by Scharlau (Barcelona, Spain). Ammonium hydroxide (NH_4_OH, 35%) was purchased from Fisher scientific (Leicestershire, UK). The tannery wastewater was collected from the effluent discharge of a local tannery industry (Prime Tanning Industry (Pvt.) Ltv K.M, 25 GT Rd, Muridke, Sheikhupura, Punjab) Pakistan. The tannery wastewater was characterized by determining various parameters such as pH, chemical oxygen demand (COD), biological oxygen demand (BOD) and suspended solids (SS).

### 2.2. List of Instruments

The analysis of Cr was carried out by an Atomic Absorption Spectrometer (Perkin Elmer Analyst 700, Waltham, MA, USA). An ultrasonicator (wt-230HTD, Seoul, Korea) was used for sample dispersion. To examine the surface morphology of the adsorbent, the scanning electron microscope (SEM) JSM 5910, manufactured by JEOL JEM-2100F (Tokyo, Japan), was utilized. The samples were scanned over a 2θ range of 0–70° at the scanning rate of 1 degree per minute. The FTIR analysis was carried out with an FTIR spectrometer (Perkin Elmer 103385, Waltham, MA, USA). The structural crystallinity of the adsorbent was studied using the XRD analysis model (X’Pert^3^ Powder-Malvern Panalytical, Almelo, The Netherlands). The XRD analysis was conducted using an advanced Bruker anode X-ray diffractometer with Cu Kα (λ = 1.5406 A) radiation. A glass electrode of model 422 WTW, Weilhmein, Germany, was used for the adjustment of the sample’s pH.

### 2.3. Synthesis of MNPs/rGO/PMMA Composite

#### 2.3.1. Synthesis of GO

GO was synthesized from powder graphite with Hummers’ technique [34]. Firstly, 2 g of graphite was slowly added to the solution of 1 g of NaNO_3_ dissolved in 92 mL of sulfuric acid (H_2_SO_4_) in the conical flak fitted in a bath containing ice, to maintain the temperature at 0 °C. KMnO_4_ (12 g) was added and stirred vigorously; the paste that formed was removed from the ice bath, further stirred for two h at 35 °C and then left to settle overnight. Deionized water (184 mL) was added to the suspension under vigorous stirring. The temperature was raised to 95 °C and the flask was cooled to 45 °C on an ice bath. About 10 mL of H_2_O_2_ was added to the reaction mixture until a brown–yellow product of GO was formed, which was collected through filtration. Further, it was washed with distilled water and diluted HCl, then dried under vacuum at 60 °C.

#### 2.3.2. Synthesis of rGO

The synthesized GO was then reduced to reduced graphene oxide (rGO) through the method reported in the literature [35]. In total, 1.0 g of GO was dispersed into 150 mL of deionized water and sonicated using an ultrasonic bath for 3 h under nitrogen atmosphere. About 5 mL of hydrazine hydrate solution was added to the dispersion and then stirred for 30 min till the color of the content changed from dark brown to dark black. The end product was washed with double-distilled water and ethanol for many times to evacuate undesirable contaminations.

#### 2.3.3. Preparation of MNPs/GO

An in situ method was used to prepare MNPs/rGO, in which 1 g of rGO was dispersed in 100 mL of distilled water and sonicated using ultrasonication for 2 h. The FeCl_3_ and FeSO_4_ solutions were added to the suspension in a ratio of 2:1 and agitated for 2 h at 80 °C under nitrogen atmosphere. A volume of 10 mL of 35% NH_4_OH solution was added dropwise for 2 h to blend. A dark-black precipitate of MNPs/rGO was obtained; the sequences of changes are indicated by the following reactions:

$${\mathrm{Fe}}^{2+}+2{\mathrm{Fe}}^{3+}+6{\mathrm{OH}}^{-}\;\to \;3\mathrm{Fe}{\left(\mathrm{OH}\right)}_{2}\;3\mathrm{Fe}{\left(\mathrm{OH}\right)}_{2\;}\to {\;\mathrm{Fe}}_{3}O_{4}+2H_{2}O_{\;}$$

**Scheme 1:**
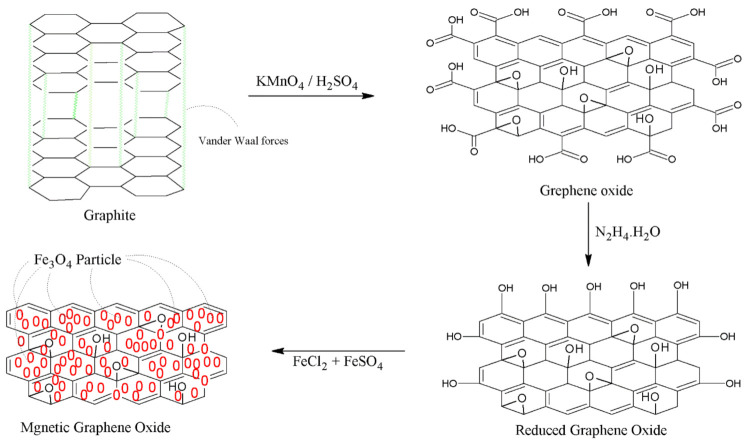
Synthesis of GO, rGO and MNPs/rGO.

The suspension was separated from the solution using an external magnetic field. It was washed several times with distilled water and ethanol to eliminate excessive base and dissociate free ions of Fe(II) and Fe(III). Then, the final product was dried under vacuum at 30 °C. The sequence of reactions is shown in Scheme 1. The ratio of FeCl_3_ and FeSO_4_ to rGO is 2:1:1, respectively.

#### 2.3.4. Preparation of MNPs/rGO and PMMA Composite

The MNPs/rGO/PMMA composite was fabricated using an in situ polymerization technique [36]. A total of 1 g MNPs/rGO was dispersed in 100 mL of deionized waster and 20 mL methyl methacrylate (MMA) was mixed to it. The suspension was dispersed through ultrasonication for 30 min. A total of 0.2 g of initiator 2,2-Azobisisobutyronitrile (ABBN) was added to the reaction mixture and heated to 70 °C, then stirred for 25 min under controlled condition. Then, a few mL of (CH_3_)_2_CO was added to the final product, decanted into a petri dish and dried at room temperature. The theoretical weight ratio of MNPs:rGO:PMMA was approximately 4:1:1.8 g.

### 2.4. Batch Adsorption and Desorption Studies

The tannery wastewater sample was first filtered with Whatman filter paper (grade 41, 20–25 µm) to remove suspended solids. In the batch mode adsorption experiments, a wastewater sample of about 10 mL containing 1640 mg/L of Cr(VI) was taken in a 50 mL conical flask. Its pH was adjusted to 2 (with the addition of 0.1 M HCl or NaOH) and 150 mg of the MNPs/rGO–PMMA composite adsorbent was added and agitated for 30 min in a shaking bath. The adsorbent was collected from the water sample by holding a magnet along the wall of the flask, then the sample was collected and subjected to analysis. The concentration of Cr(VI) ions was determined via flame atomic absorption spectrometry (FAAS).

**Scheme 2:**
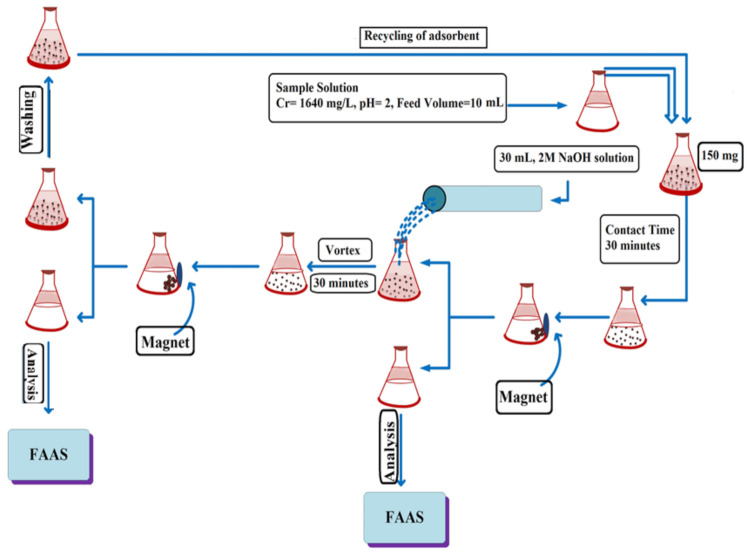
Batch adsorption and desorption studies.

In the desorption experiments, the composite sample was shacked with 2 M sodium hydroxide (NaOH) for 30 min to desorb Cr(VI) ions from the surface. The composite was collected from the medium by an external magnetic field. The eluent was also analyzed via FAAS to determine the Cr(VI) concentration. The scheme of the experiment is shown below (Scheme 2).

The batch mode adsorption of Cr(VI) onto the MNPs/rGO/PMMA was investigated in triplicate and the average values of the standard deviations were recorded. The percent adsorption of Cr was determined using Equation (1)

(1) $$\%,\;\mathrm{Adsorption}=\frac{C_{0}-C_{t}}{C_{0}}\times 100$$

where “C_0_” (mg·L^−1^) is the original Cr concentration in the wastewater sample, while “C_t_” (mg·L^−1^) is the final Cr concentration in the outlet sample. The adsorption capacity (q_e_) (the amount of adsorbate (mg·L^−1^) held per mass of adsorbent (mg)) was determined using Equation (2)

(2) $$q_{e}=\frac{C_{0}-C_{t}}{m}\times v$$

where “m” is the adsorbent mass (g), while “V” (mL) is the volume of the sample solution. The batch experiments were repeated for the amount of adsorbent to calculate the maximum adsorbent dose. Various temperatures were applied, ranging from 293 K to 393 K, to calculate the entropy change (∆S^o^), Gibbs free energy (Δ*G*^o^) and enthalpy change (∆H^o^). Then, the pH was optimized to describe the pH dependence of the adsorption process. Similar batch tests were directed for sample volume to investigate the maximum sample volume. The adsorption kinetics was calculated from the vortex time, running from 10 min to 60 min. 

### 2.5. Fixed-Bed Column Experiments

**Scheme 3:**
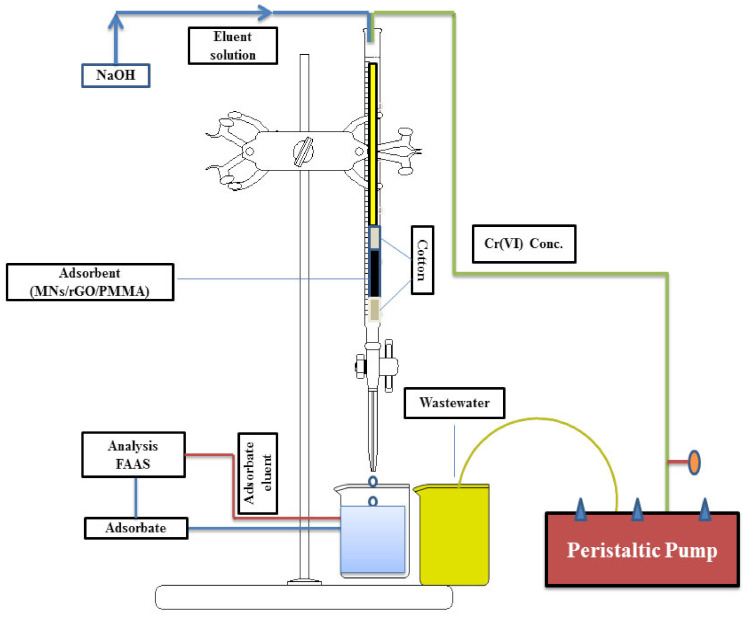
Adsorption and desorption scheme of fixed-bed column.

For fixed-bed column adsorption studies, a glass column of 50 cm in height and 2 cm in internal diameter was used, into which a swab of glass wool was inserted. A total of 1 g of MNPs/rGO/PMMA adsorbent was loaded into the column and another glass wool swab was placed on the top of the adsorbent bed to avoid disturbance of the adsorbent surface during the flow of the sample. The diagrammatic illustration of the fixed-bed column is shown in Scheme 3. Initially, the composite packed column was rinsed with distilled water and left for a night to clarify the thorough distribution of adsorbent particles. Wastewater containing 1640 mg/g was impelled, downstream mode, into the column with the assistance of a peristaltic pump. Primarily, the collected adsorbate was discarded for one minute to ensure an unbroken stream rate. Afterward, the discharge samples were collected successively for further analysis at consistent interval of time, flow rate and adsorbent masses. This was performed from the bottom of the column to investigate the breakthrough point.

The continuous fixed-bed column process was stopped when the effluent concentration was equal to the initial concentration. The breakthrough cure was calculated from different adsorbent masses and flow rates by plotting graphs between C_t_/C_0_ and time. In most of the continuous fixed-bed system, the breakthrough curves are S-shaped with variable degrees of sharpness and location of the breakthrough point.

To regenerate the adsorbent and recover the Cr(VI) ion, an eluent reagent was used. The Cr(VI) ions which were loaded on different masses of adsorbent with a uniform flow rate 2 mL/min were desorbed using a 2 M NaOH solution. Similarly, the Cr(VI) analytes which were loaded on 3 g of composite with different flow rates (1, 2 and 3 m/L/min) were also desorbed through the 2M NaOH solution.

## 3. Results and Discussions

### 3.1. Characteristics of Adsorbent

**Figure 1:**
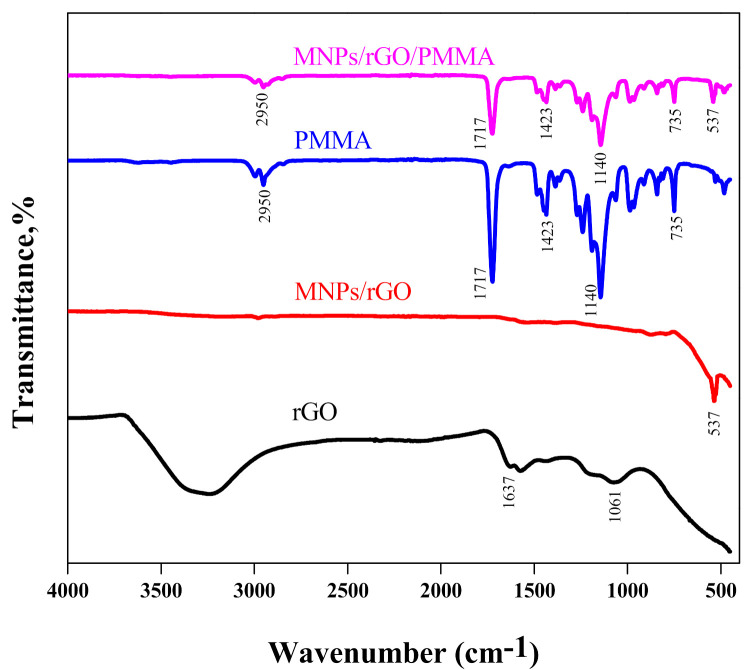
FTIR spectra of rGO, Fe_3_O_4_/rGO, PMMA and MNPs/rGO/PMMA.

The synthesized composite (MNPs/rGO/PMMA) was confirmed via FTIR analysis, XRD analysis and SEM analysis. Figure 1 shows the FTIR spectra of rGO, MNPs/rGO, PMMA and MNPs/rGO/PMMA. The FTIR spectrum of rGO shows characteristic peaks of the C=C stretching bond of alkene and C–O stretching of the hydroxyl group at “1637 cm^−1^” and “1061 cm^−1^”, respectively. The rGO (rGO) impregnated with magnetic Fe_3_O_4_ showed an additional peak at “537 cm^−1^”, which gives a clear identification of the Fe–O–Fe bond [37]. The FTIR spectrum of PMMA displays the characteristics peaks of C–H bending of the α– methyl group, C–O stretching of aliphatic ether, C–H bending of a methylene group, C=O stretching of the carboxylic functional group and C–H stretching of the methyl group at 735 cm^−1^, 1140 cm^−1^, 1423 cm^−1^, 1717 cm^−1^ and 2950 cm^−1^, respectively. The spectrum of the composite sample reflects the extra peak of Fe–O–Fe stretching at 537 cm^−1^ due to the magnetic rGO (MNPs/rGO) incorporated to PMMA, which indicates that the polymer was successfully magnetized [38].

**Figure 2:**
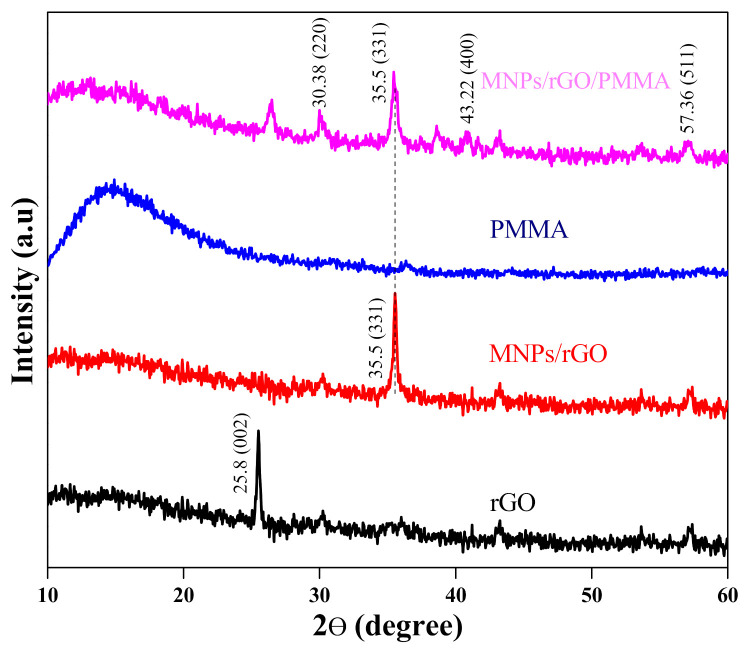
XRD spectra of rGO, Fe_3_O_4_/rGO, PMMA and MNPs/rGO/PMMA.

The XRD configuration of rGO indicates a solid peak at 2θ of 25.8, which confirms the reduction of GO as shown in Figure 2 [39]. In the case of magnetized rGO, a peak at 2θ of 35.5 displayed that it matched with a face-centered cubic crystal of JCPD file card number 19-0629, suggesting the confirmation of impregnation of magnetic Fe_3_O_4_ on the rGO [37]. The XRD arrangement of pure PMMA gives no peak due to its amorphous nature. The XRD design of the composite (MNPs/rGO/PMMA), exhibits peaks corresponding to Fe_3_O_4_ at 2θ of 30.38° (220), 35.5° (331), 43.22° (400) and 57.36° (511), respectively, which confirms the MNPs/rGO/PMMA composite [40,41]. From the XRD data, the crystallite size of the composite was estimated to be 31 nm. 

**Figure 3:**
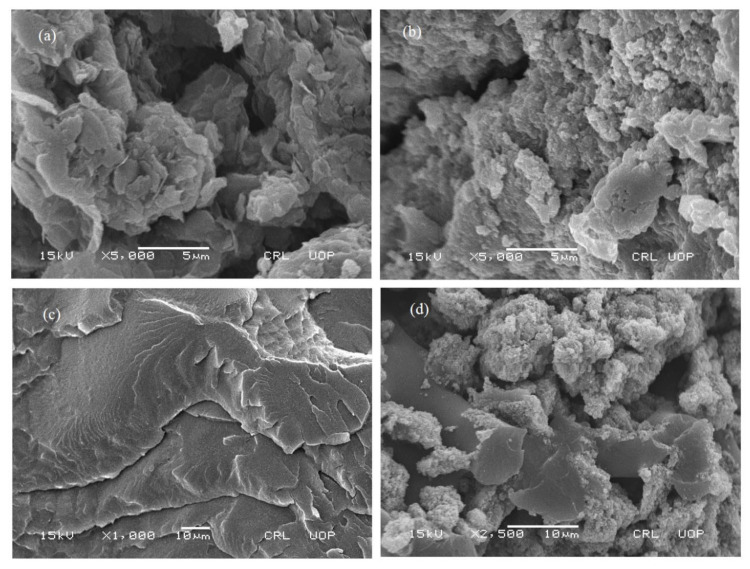
SEM of (**a**) rGO, (**b**) Fe_3_O_4_/rGO, (**c**) PMMA and (**d**) MNPs/rGO/PMMA.

The SEM micrographs of rGO, MNPs/rGO, PMMA and MNPs/rGO/PMMA are given in Figure 3a–d. The micrograph of rGO indicates a fluffy and chipped morphology, which may be a stacked structure of graphene sheets linked by Van der Waal’s forces, as shown in Figure 3a. Fe_3_O_4_-NPs were consistently scattered over the rGO sheets, as shown in Figure 3b [42]. The PMMA matrix is in the form of smooth uniform layers with no pores, in the form of sheets spread over each other, as can be seen in Figure 3c [43]. The micrograph of MNPs/rGO/PMMA confirms the magnetic MNPs dispersed on the surface of the PMMA/rGO sheets. MNPs/rGO/PMMA seems a uniform layer, indicating all components were properly incorporated together (see Figure 3d) [44]. From the SEM micrographs, the average size of the rGO flakes is found to be about 2000 nm, whereas that of MNPS is about 100 nm.

### 3.2. Physiochemical Characterization of Wastewater

Previously, to bring the tannery wastewater into batch and column modes of adsorption, the sample was evaluated for a few limits, e.g., COD, biological oxygen demand (BOD), pH, Cr concentration and suspended solids. The Cr concentration in wastewater was found to be “1640” mg·L^−1^, COD was “1130” mg·L^−1^, while BOD was calculated at about “396” mg·L^−1^. Similarly, suspended solids in the sample were calculated to be “960” mgL^−1^ and a pH of 3.17 was observed. As indicated by the Environmental Health Safety Guidelines (EHSG), the possible edge for COD and pH in wastewater are 150 mg·L^−1^ and 6–9, respectively. Likewise, the permissible level of Cr in industrial wastewater, according to the Environmental Protection Agency (EPA), is “200” mg·L^−1^. Thus, it is obvious, from the above proof, that these limits do not follow the possible edges for wastewater, especially if the Cr concentration is extremely high.

## 4. Adsorption Experiment

The adsorption of Cr(VI) over the MNPs/rGO/PMMA was carried out in batch mode under different conditions, including pH, adsorption time, adsorbent dose, temperatures, etc.

### 4.1. pH Investigation

**Figure 4:**
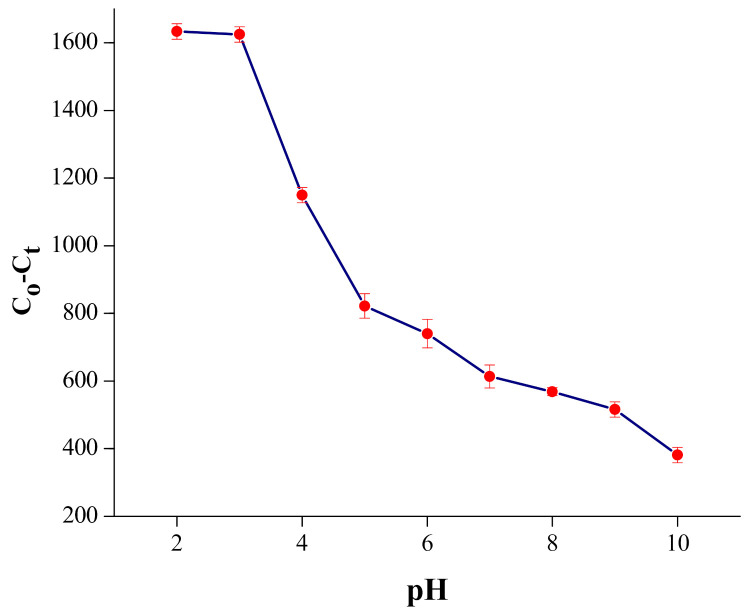
Effect of pH on adsorption of Cr(VI) on the MNPs/rGO/PMMA composite; adsorbent amount, 150 mg; shacking time, 30 min.

The pH of the reaction medium has an exceptional impact on the charge density on the adsorbent surface and the assurance of the change of Cr species [45]. The Cr(VI) adsorption on the composite was carried at various pH ranges, from 2 to 10, while the other parameters were kept constant. The pH of the investigated solution was adjusted using 0.1 N NaOH or HCl solution, according to necessity. It was noticed that the most extreme adsorption was found at pH 2.0 (100 ± 1), as shown in Figure 4. 

The maximum adsorption of Cr at this pH was observed due to the existence of Cr(VI) in various forms, such as dihydrogen chromate (H_2_CrO_4_), hydrogen chromate (HCrO_4_^−^), hydrogen dichromate (HCr_2_O_7_^−^), dichromate (Cr_2_O_7_^2−^) and chromate (CrO_4_^2−^) [46]. The dominancy of all above ions greatly depends on the pH solution, as well as on the total Cr concentration. At pH from 2 to 6, hydrogen chromate (HCrO_4_^−^) and dichromate (Cr_2_O_7_^2−^) are the dominant species. Dihydrogen chromate (H_2_CrO_4_) is the main species at low pH values (less < 1) [47]. The dominant type of Cr(VI) at pH 2 is hydrogen chromate (HCrO_4_^−^), generated from the dichromate (Cr_2_O_7_^2−^) based in reaction below [48].

$${\mathrm{Cr}}_{2}O_{7}^{2-}+{\;H}_{2}O\;\to 2{\mathrm{HCrO}}_{4}^{\_}$$

The Cr_2_O_7_^2−^ particle holds two negative charges; hence, it needs two active sites for successful adsorption, while hydrogen chromate (HCrO_4_^−^) particles need one active site of the adsorbent (MNPs/rGO/PMMA) for successful adsorption [49]. Therefore, the adsorption capacity of the (HCrO_4_^−^) is twice that of (Cr_2_O_7_^2−^). Likewise, at low pH, the surface of adsorbent became protonated due to the availability of a large number of H^+^ ions, which creates an electrostatic force within a protonated functional group of adsorbent and Cr anions. The adsorption rate falls quickly and spans to 38% at pH 6, above pH 3. This is most likely because of the general change in surface charge on the adsorbent and, subsequently, adsorption decline. At pH > 8, the percent adsorption was further decreased because of the double resistance of two anions (CrO_4_^2−^ and OH^−^) on the surface of the adsorbent [50].

### 4.2. Adsorbent Dose Investigation

**Figure 5:**
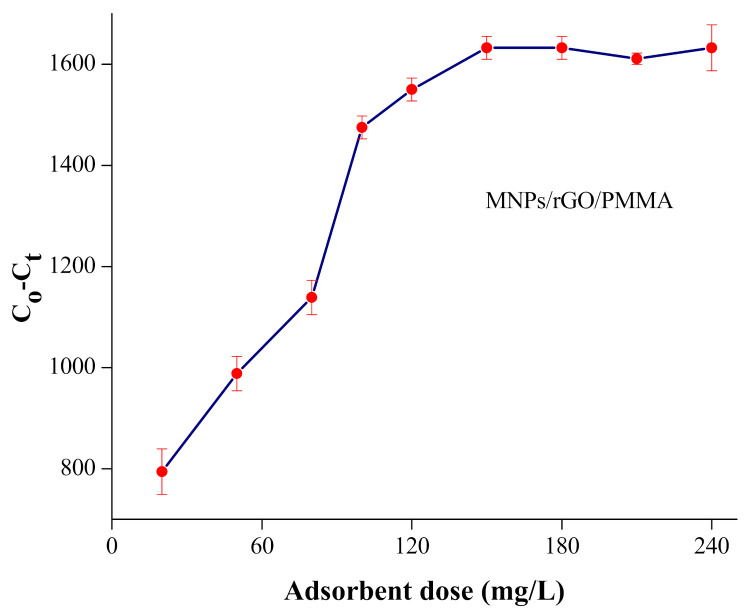
Effect of adsorbent amount on adsorption of Cr(VI) on MNPs/rGO/PMMA composite; pH 2; sample volume, 10 mL; shaking time, 30 min.

The percent adsorption of Cr(VI) is dependent on the amount of adsorbent dose in wastewater. To explore the impact of adsorbent (MNPs/rGO/PMMA) dose, batch adsorption experiments were conducted using dosages ranging from 20 mg to 250 mg. Results indicate that the % Cr(VI) adsorption was enhanced, as the amount of adsorbent dose was increased, and the maximum percent adsorption was found at 150 mg (99.53 ± 1), as shown in Figure 5. The significant rise in Cr(VI) adsorption with the increase in the amount of composite material was due to the availability of a larger surface region of the adsorbent and a large number of active sites [51]. A further enhancement of the amount of composite material to 240 mg showed no considerable variation in percent adsorption of Cr(VI). Hence, 150 mg was chosen as an ideal amount of (MNPs/rGO/PMMA) adsorbent to the be studied.

### 4.3. Kinetic Investigation

**Figure 6:**
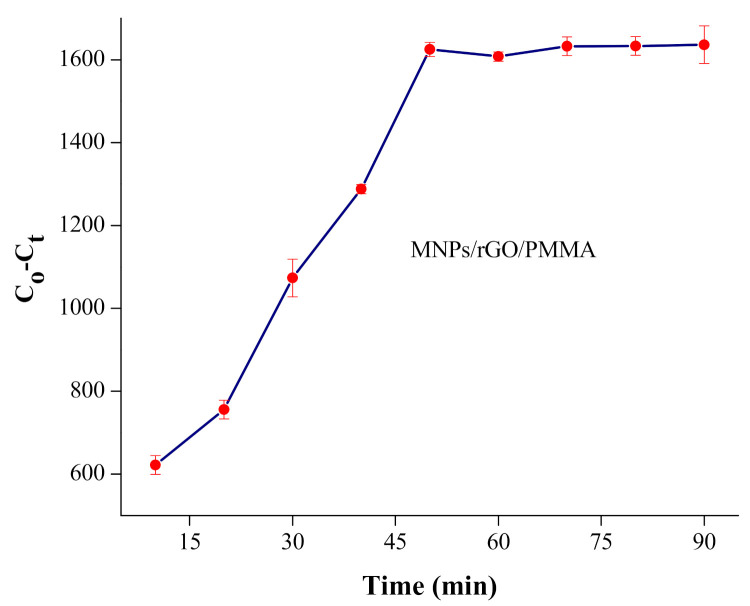
Effect of shaking time on adsorption of Cr(VI) on MNPs/rGO/PMMA composite; adsorbent amount, 150 mg; sample volume, 10 mL; pH 2.

To achieve maximum adsorption over adsorbent (MNPs/rGO/PMMA) versus time, various contact periods of time, running from 10 min to 1 h, were determined. The outcomes show that the % adsorption of Cr(VI) was enhanced versus time; finally, stability was reached after 30 min, as shown in Figure 6.

A further increase in shacking time caused no considerable change in adsorption. Thus, 30 min of contact time was chosen to attain the most extreme adsorption for each batch experiment.

To clarify the systemic adsorption procedure, the shacking data were applied to pseudo-1st order and pseudo-2nd order kinetics models using Equations (3) and (4), respectively.

(3) $$\log\left(q_{e}-q_{t}\right)={\mathrm{logq}}_{e}-\frac{K_{1}}{2.303}t$$

(4) $$\frac{t}{q_{t}}=\frac{t}{q_{e}}+\frac{1}{k_{2}q_{e}{}^{2}}$$

where “k_1_” (1/min) is the proportionality constant of pseudo-1st order model, while “K_2_” (g^−1^·mg^−1^·min^−1^) is the proportionality constant of pseudo-2nd order model; “q_t_” (mg·g^−1^) is the quantity of metal ions (mg) adsorbed per unit quantity of adsorbent (g) at any time (t) and “q_e_” (mg·g^−1^) is the quantity of metal ions (mg) adsorbed per unit quantity (g) of adsorbent at dynamic equilibrium.

Various kinetic factors of pseudo-1st order and pseudo-2nd order kinetics were determined from the slope and intercept, as displayed in Figures S1 and S2 (Supplementary Materials), respectively. The calculated and experimental results are listed in Table 1. The value of “R^2^” for pseudo-1st order kinetic was calculated to be 0.86, which is smaller than those of pseudo-2nd order kinetic (0.98). The value of adsorption efficiency (q_e_) for the pseudo-1st order of kinetic was calculated to be 23.93 mg g^−1^, which is smaller than the experimental adsorption efficiency, 109.33 mg g^−1^, whereas the calculated value of adsorption capacity (q_e_) for pseudo-2nd order kinetic was 166.66 mg g^−1^, which is higher than the experimental value of 109.33 mg g^−1^.

Similarly, the value of K_1_ was 0.151, which is lower than that of K_2_ (0.362), suggesting that the Cr(VI) adsorption process follows the pseudo-2nd order kinetic model.

### 4.4. Adsorption Isotherms

To calculate various adsorption parameters, the data obtained from the adsorption process were fitted to the Langmuir isotherm and Freundlich isotherm. The Langmuir adsorption isotherm is expressed by Equation (5):

(5) $$\frac{C_{e}}{q_{e}}=\frac{1}{q_{m\;}k_{b}}+\frac{1}{q_{{m\;C}_{e}}}$$

In the above expression, “C_e_” (mg·L^−1^) is the equilibrium adsorption concentration and “q_e_” (mg·g^−1^) is the quantity of Cr(VI) ions adsorbed at equilibrium state. Similarly, “q_m_” (mg·g^−1^) is the highest adsorption capacity and “K_b_” (Langmuir constant) is the constant associated with energy. R_L_ (dimensionless isolating component) is determined from the Langmuir isotherm using Equation (6):

(6) $$R_{L}=\frac{1}{1+k_{b}C_{e}}$$

The projected value of “R_L_” verifies whether the adsorption is favorable, unfavorable, reversible, or irreversible. If the R_L_ value is more than one (1 < R_L_), the adsorption is unfavorable. Similarly, when the value of R_L_ is equivalent to one (R_L_ = 1), it indicates that the adsorption process is reversible. If the R_L_ value is equivalent to zero (R_L_ = 0), it denotes that the adsorption process is irreversible. R_L_ values of 0 < R_L_ < 1 suggest that the adsorption process is favorable [52]. The slope and intercept values that were achieved from the Langmuir adsorption isotherm graph verified that the adsorption process may have been unfavorable or favorable, irreversible, or reversible, as indicated in Table 2. The binding constant and adsorbent capacities were calculated from the plot between C_e_/q_e_ and C_e_, as indicated in Figure S3 (Supplementary Materials). The results suggest that the adsorption capacity (q_m_) of 240.96 mg·g^−1^ was investigated. The dimensionless separating constant (R_L_) value was found to be 0.199, the coefficient correlation factor (R^2^) value was 0.991, while a Langmuir constant (K_b_) value of 4.021 was achieved.

To examine the adsorption information through the Freundlich adsorption isotherm, Equation (7) was used:

(7) $${\mathrm{logq}}_{e}=\mathrm{logKf}+\frac{1}{n}{\mathrm{logC}}_{e}$$

The slope and intercept values obtained from the Freundlich adsorption isotherm graph concluded whether the adsorption was favorable or unfavorable. It also explained whether the system was heterogeneous or not, as shown in Figure S4 (Supplementary Materials). A Freundlich isotherm parameter such as 1/n gives useful evidence about the adsorption system. A value of 1/n > 1 suggests that the system is imperfect for low concentrations but thrives at high concentrations. Likewise, when the value of 1/n is smaller than one (1/n < 1), it means that the adsorption system is perfect over the whole range of concentrations. Correspondingly, if the 1/n value is equal to one (1/n = 1), it suggests that the adsorption system is homogenous [53].

In the current study, the value of R^2^ (0.99124) showed that the Langmuir adsorption isotherm fitted better to the adsorption data than the Freundlich isotherm (R^2^ = 0.97215). On the other hand, the R_L_ value was 0.1999, which means that the adsorption of Cr(VI) on MNPs/rGO/PMMA was favorable and monolayer. In addition, the value of 1/n was found to be 0.35102, which confirms that the adsorption was positive at all possible concentrations and the adsorption framework was heterogeneous.

### 4.5. Thermodynamic Investigation

**Figure 7:**
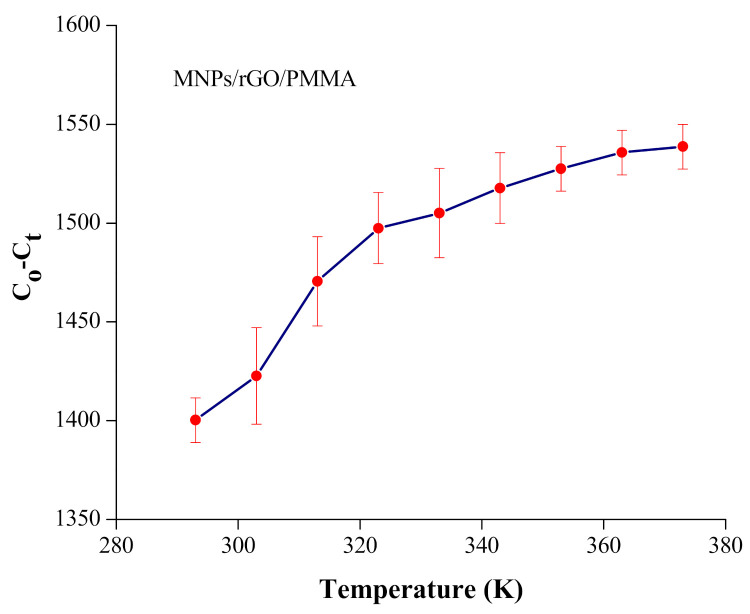
Effect of temperature on adsorption of Cr(VI) on MNPs/rGO/PMMA composite; adsorbent amount, 150 mg; shacking time, 30 min; pH 2.

To investigate the adsorption system versus temperature, the influence of temperature on the adsorption of Cr(VI) was achieved at various temperatures, from 293 to 373 K, at optimized conditions, as illustrated in Figure 7.

The adsorption of Cr(VI) increased proportionally with the increase in temperature from 293 to 373 K. This can be attributed to the fact that a temperature increase leads to a corresponding increase in the system’s energy, thereby providing more chances of adsorbate-active site interactions. As the temperature was increased, there might have been an increase in the number of active sites, thereby rupturing the functional group bond occurred on the surface of the adsorbent, which improves the adsorption proficiency [50]. The thermodynamic parameters, including Gibbs free energy (ΔG^o^), enthalpy (ΔH^o^) and entropy (ΔS^o^), were determined using Equations (8)–(10), respectively.

∆G^o^ = −RT ln K_D_ (8)

(9) $$\text{∆}H^{o}=R\;\frac{T_{2T_{1}}}{\;\;T_{2-T_{1}}}\ln\;\frac{K_{2}}{K_{1}}$$

(10) $$\text{∆}S^{o}=\frac{\left(∆H^{o}-∆G^{o}\right)}{T}$$

The negative values of ΔG^o^ indicated the spontaneous nature of Cr(VI) adsorption. Similarly, ΔG^o^ values became more negative as the temperature increased, which shows that the adsorption process was improved as the temperature was enhanced. The ΔH^o^ and ΔS^o^ values were determined from the plot shown in Figure S5 (Supplementary Materials). The positive value of ΔH^o^ demonstrates that the current adsorption system was endothermic in nature. Besides, the positive value of ΔS^o^ confirms that the adsorption system was spontaneous, as shown in Table 3.

### 4.6. Volume of Eluent Optimization and Desorption Studies

The desorption of metal particles from the adsorbent is an important aspect in wastewater treatment. For this purpose, Cr(VI) was desorbed from MNPs/rGO/for 30 min with (1 and 2 M) solutions of NaOH and (1 and 2M) NH_4_OH, respectively. As the desorption process was completed, the filtrate was isolated from the adsorbent utilizing the outer magnetic field and was then analyzed through the atomic absorption spectrometer. The results indicate that the greatest desorption of Cr(VI) (95 ± 1) from the adsorbent was achieved with the 2M NaOH solution, while, with NH_4_OH, the maximum desorption attained was 78 ± 1, as displayed in Table 4. 

**Figure 8:**
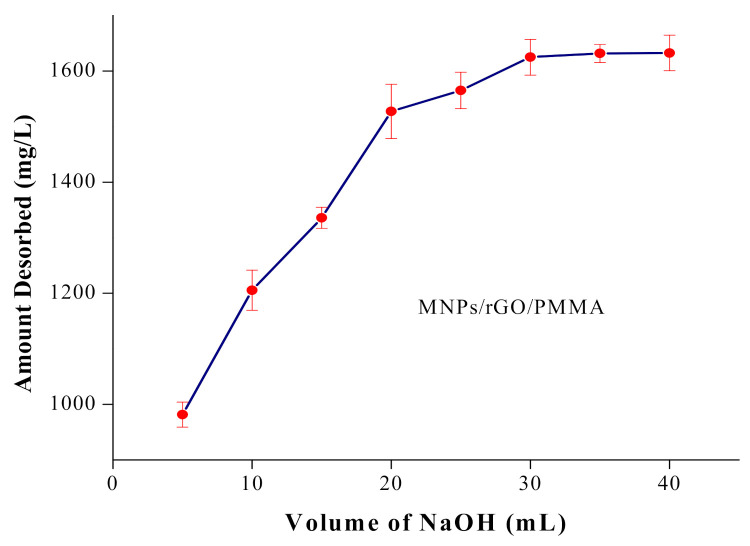
Effect of eluent volume on % recovery of Cr(VI) from MNPs/rGO/PMMA composite.

The desorption process for Cr(VI) from the adsorbent with alkaline solutions has been well documented [54]. The volume of NaOH solution (10–40 mL) revealed that, when the volume eluent reagent increased, the percent desorption increased and the equilibrium was established up to 30 mL. Hence, 30 mL of 2 M NaOH was suggested for the Cr(VI) desorption, as demonstrated in Figure 8.

### 4.7. Recovery of Cr(VI), Recycling and Regeneration of the Adsorbent

**Figure 9:**
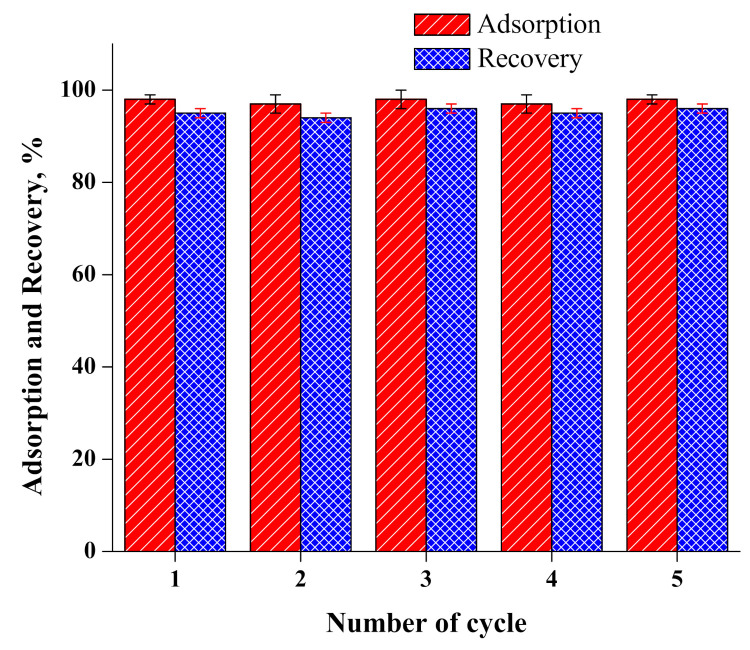
Recycling of MNPs/rGO/PMMA composite; eluent, 2M NaOH solution; volume, 30 mL.

The adsorbent’s reuse and recovery of Cr(VI) are important parameters in wastewater treatment in terms of minimizing process costs. In the current examination, recycling experiments were conducted following the adsorption and desorption of Cr(VI) at optimized conditions. After adsorption, desorption was performed using 30 mL of NaOH solution (2M) shacked for 30 min. As the desorption process was finished, the MNPs/rGO/PMMA composite was washed with double distilled water as well as dilute HCl (0.1M) and dried under vacuum at 60 °C for reuse. The results of marginal loss in the adsorption of Cr(VI) after five repeated cycles indicate the highly stable nature of the newly designed MNPs/rGO/PMMA (Figure 9). During the desorption process, the recovery of Cr(VI) from the adsorbent was 95%, 94%, 96%, 95% and 96% in each cycle, respectively. The current investigation was not assisted to control the water contamination caused by Cr(VI) effluents. At the same time, it is vital to recover the industrially important Cr metal from industrial effluents which might be reused or recycled in different applications.

The selectivity of the MNPs/rGO/PMMA composite towards the adsorption of Cr(IV) in the presence of various competing ions was investigated, in batch mode adsorption with various salts at different concentrations. The results show that, in the presence of various ions, the removal of Cr(VI) was minimally affected, indicating the high selectivity of the composite adsorbents towards Cr(VI), as indicated in Table 5.

## 5. Theoretical Analysis of Fixed-Bed Column Data

The quantity of Cr(VI) adsorbed on MNPs/rGO/PMMA was intended from the region under the breakthrough curve using Equation (11):

(11) $$q_{\mathrm{total}}=\frac{Q}{1000}\int_{t=0}^{t=\mathrm{total}}C_{\mathrm{ad}}\mathrm{dv}$$

where “Q” is the flow rate (mL/min), “t” is the time of total flow (min) and “C_ad_” is the adsorbed concentration of Cr(VI) mg/L. The adsorption capacity of the MNPs/rGO/PMMA was calculated from the breakthrough curve using Equation (12):

(12) $$q_{e=}\frac{C_{0}}{m}\int_{0}^{\mathrm{vb}}\left(1-\frac{C_{t}}{C_{0}}\right)\mathrm{dv}$$

where “q_e_” is the adsorption capacity of MNPs/rGO/PMMA at the breakthrough curve (mg/g), “m” is the mass of the adsorbent (g), “C_t_” is the outlet solution concentration (mg/L) at any time (min), “C_0_” is the initial concentration (mg/L) and “V” is the volume (L) of the treated solution. Similarly, Equation (13) was used to calculate the empty bed residence time of the continuous bed column:

(13) $$\mathrm{Empty}\;\mathrm{ed}\;\mathrm{residence}\;\mathrm{time}\;\left(\mathrm{EBRT}\right)=\frac{\mathrm{Bed}\;\mathrm{voume}}{\mathrm{volumetric}\;\mathrm{flow}\;\mathrm{rate}\;\mathrm{of}\;\mathrm{the}\;\mathrm{liquid}}$$

During the continuous process, the MNPs/rGO/PMMA adsorbent was frequently exhausted and was calculated using Equation (14):

(14) $$\mathrm{Adsorbent}\;\mathrm{exhausted}\;\mathrm{rate}\;\left(\mathrm{AER}\right)=\frac{\mathrm{Mass}\;\mathrm{of}\;\mathrm{adsorbent}\;\left(g\right)\mathrm{in}\;\mathrm{column}}{\mathrm{Volume}\;\mathrm{of}\;\mathrm{water}\;\mathrm{treated}}$$

The bed volume (BV) of the continuous fixed bed adsorbent in the column was obtained through Equation (15):

(15) $$\mathrm{Bed}\;\mathrm{volume}=\frac{\mathrm{volume}\;\mathrm{of}\;\mathrm{water}\;\mathrm{treated}\;\mathrm{at}\;\mathrm{breakthrough}\;\mathrm{curve}\;\left(L\right)}{\mathrm{volume}\;\mathrm{of}\;\mathrm{adsorbent}\;\mathrm{bed}}$$

The proficiency of the continuous fixed-bed column was studied under various flow rates and adsorbent masses. Likewise, the fixed-bed adsorption in the column was also applied to mathematical models, including the Thomas and Yoon–Nelson Models.

### 5.1. Effect of Mass of MNPs/rGO/PMMA Composite on the Breakthrough Curve

**Figure 10:**
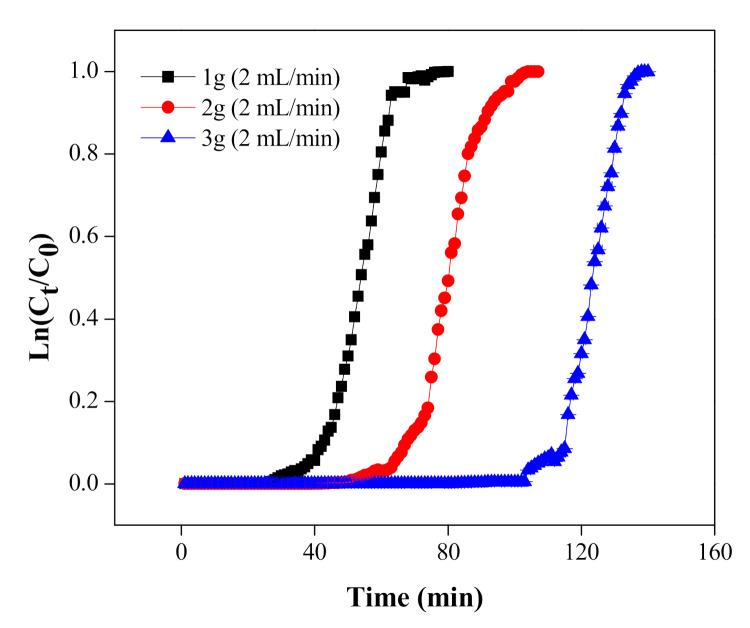
Cr(VI) adsorption breakthrough with different adsorbent masses.

In order to investigate the impact of adsorbent masses on the breakthrough curve, tannery wastewater (Cr(VI), 1640 mg/L) was passed through various adsorbent masses extending from 1 g to 3 g, while other parameters, such as flow rate, pH and concentration of Cr(VI), were kept constant. The results in Figure 10 display that the breakthrough time was enhanced as the adsorbent mass was increased from 1 g to 3 g. This could be ascribed to the availability of more active sites of MNPs/rGO/PMMA for the adsorption of Cr(VI) with the increase in mass [55].

It might be induced from the results that the breakthrough curve is dependent on the MNPs/rGO/PMMA bed mass. It was found that the former breakthrough curve was seen at lower adsorbent mass and, after the breakthrough, a sharp ascent occurs in the Cr concentration. The sharp ascent is due to the exit of the mass exchange zone; at that point, the bed has an insignificant capacity to adsorb Cr(VI) [56]. At the same time, the adsorption capacity (q_e_) and adsorbent exhaustion rate (AER) were calculated from the breakthrough curve and are given in Table 6.

The adsorption capacity was enhanced with an increase in the adsorbent mass due to the increase in the surface zone, which encouraged the availability of active sites for adsorption. Similarly, the obtained values of the adsorbent exhaustion rate (AER) declined from 20.00 g/L to 14.56 g/L with an increase in the adsorbent masses from 1 g to 3 g. The lower values of the adsorbent exhaustion rate (AER) showed better execution for adsorption [57].

### 5.2. Effect of Flow Rate

**Figure 11:**
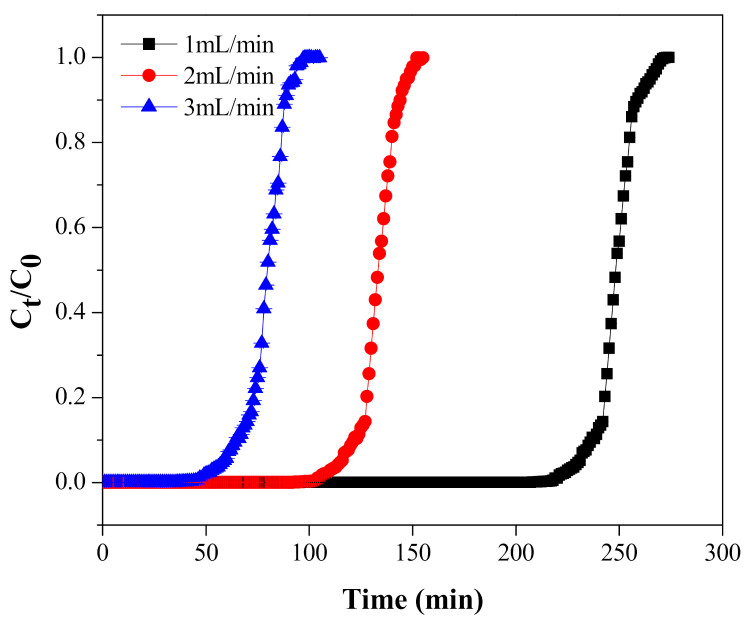
Cr(VI) adsorption breakthrough at different flow rates.

The flow rate of the wastewater stream plays a substantial role in estimating the execution of the adsorption process, especially at the industrial level, to achieve maximum treatment of the influent. Therefore, the impact of stream rate on the adsorption of Cr(VI) by MNPs/rGO/PMMA was studied at different flow rates ranging from 1 mL/min to 3 mL/min, keeping the other parameters constant. The results are shown in Figure 11 and the calculated process parameter values are recorded in Table 5. As shown in Figure 11, the breakthrough curve shortened as the flow rate was enhanced from 1 mL/min to 3 mL/min, for constant adsorbent mass. 

This is presumably due to the increased mass passage rate, which brings a reduction in the time needed to obtain the preferred breakthrough concentration [58]. Additionally, the adsorption capacity and ARE also declined from 120.06 to 75.01 mg/L and from 11.02 to 9.08 mg/L, respectively. This could be for the reason that, at a higher stream rate, the adsorbent in the fixed-bed column rapidly saturated and there was insufficient contact time for the complete adsorption of Cr on the adsorbent in the fixed-bed column [46].

### 5.3. Desorption and Regeneration of the Column

**Figure 12:**
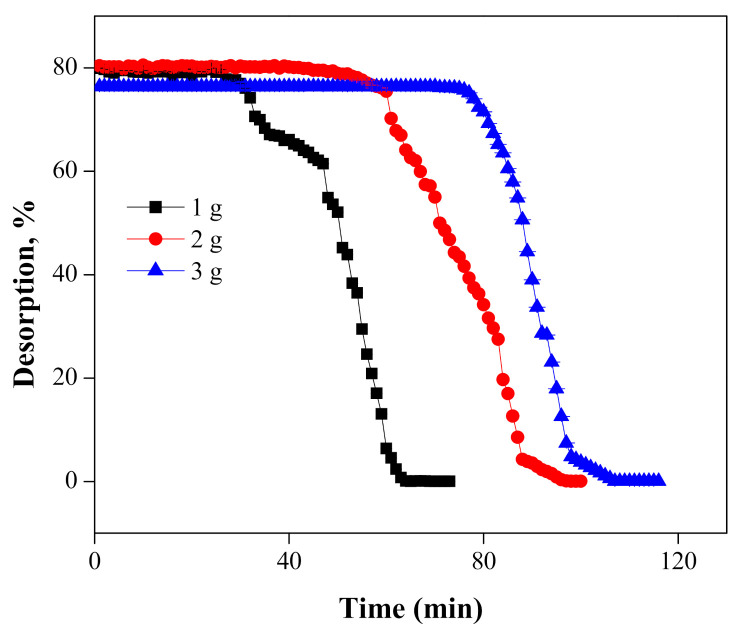
Desorption of Cr(VI) at various adsorbent masses using the 2 M NaOH solution.

During the desorption experiments, Cr(VI) was leached from a column with various masses of the MNPs/rGO/PMMA composite at a constant flow rate (2 mL/min) of the 2 M NaOH solution. The results show that the breakthrough point for desorption was found in three distinctive times, i.e., 27, 42 and 75 min, for 1, 2 and 3 g of MNPs/rGO/PMMA masses, respectively, as shown in Figure 12. The maximum recovery of Cr(VI) attained was 79.93 ± 0.07%, 80.40 ± 07% and 76.33 ± 0.008%, when loaded with 1, 2 and 3 g MNPs/rGO/PMMA at a 2 mL/min flow rate of the 2 M NaOH solution, respectively. A further increase in time caused the concentration of leached Cr(VI) to decrease the in outlet solution due to the unsaturation of Cr(VI) on the MNPs/rGO/PMMA composite [59].

**Figure 13:**
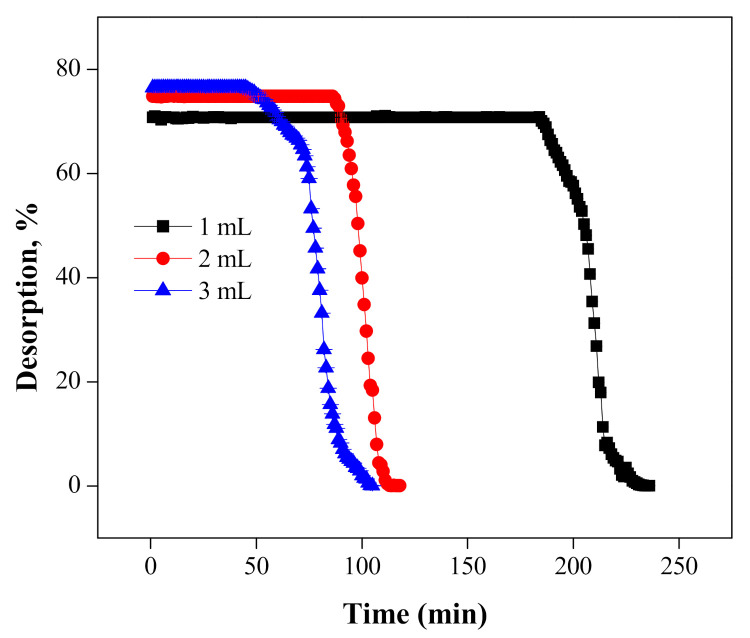
Desorption of Cr(VI) at various flow rates using the 2 M NaOH solution.

Meanwhile, the effect of various flow rates, i.e., 1, 2 and 3 mL/min was also investigated at the constant adsorbent mass of 3 g. The results are displayed in Figure 13. We observed that, as the flow rate of the NaOH solution was increased (from 1 to 3 mL/min), the desired time for the recovery of Cr(VI) decreased from 186 to 47 min. The maximum recovery of Cr(VI) in each column was observed to be 70.77 ± 0.07, 74.82 ± 0.04 and 76.45 ± 0.001, with 1, 2 and 3 mL/min flow rates of NaOH. 

### 5.4. Theoretical Modeling of the Breakthrough Curve

The theoretical calculation of the column studies was assessed by the Thomas and Yoon–Nelson models to investigate the efficiency of the fixed-bed adsorption column. 

#### 5.4.1. Thomas Model

The Thomas model is a general theoretical model utilized for fixed-bed column data analysis and for the prediction of breakthrough points. The Thomas model is described on the supposition of pseudo-2nd order kinetic and Langmuir adsorption isotherm models. The Thomas model is expressed in Equation (16):

(16) $$\ln\frac{C_{0}}{C_{t}-1}=\frac{K_{\mathrm{TH}}\;}{Q}{(\mathrm{qe})(m)-K}_{\mathrm{TH}}C_{0}t$$

where “C_0_” (mL/L) and “C_t_” (mL/L) are the initial and final concentration, respectively, “q_e_” (mg/g) is the adsorbent capacity, “KTH” (mL/min g) is the Thomas model constant and “t” (min) is the total flow time. The values of “q_e_” and “K_TH_” were obtained from the linear plot between $(\ln\frac{C_{0}}{C_{t}-1}$) and time (t), as shown in Figure S6 (Supplementary Materials).

Different values of KTH and q were obtained from the Thomas model for various flow rates, as shown in Table 7, which suggests that the values of q and KTH decreased as the flow rate increased from 135.31 to 111.48 mg/g and from 11.58 × 10^−2^ to 8.82 × 10^−2^, respectively. This may be due to the short time for salute particles to adsorb on the MNPs/rGO/PMMA composite and permitted Cr(VI) before the complete adsorption. The Thomas model exhibited good fit to the adsorption data with the R2 values of 0.999, 0.993 and 0.972 under the three flow rates, respectively. From the above results, it was concluded that there are some experimental points and prediction positions which recommend the fitting of the Thomas model on the experimental breakthrough curve. The same result was found by Shalini et al.; the adsorption capacity of Cr(VI) on chemically modified Lagerstroemia speciosa bark in the fixed-bed column decreased with the increase in the flow rate [60].

#### 5.4.2. Yoon–Nelson Model

Yoon–Nelson developed a simple mathematical model for fixed-bed column adsorption and is based on the fact that the probability of adsorption rate decreases for each adsorbate molecule, which is proportional to the probability of adsorbate adsorption and the probability of adsorbate breakthrough on the adsorbent. The Yoon–Nelson model is mathematically expressed by Equation (17):

(17) $$\ln\;\left(\frac{C_{t}}{C_{0}-C_{t}}\right)={\;K}_{\mathrm{YN}}t-K_{\mathrm{YN}}\tau$$

where “C_0_” (mg/g) and “C_t_” (mg/g) are the initial and final concentration in the outlet, “KYN” (min^−1^) is the rate constant of Yoon–Nelson and “τ” (min) is the flow time required for the 50 percent breakthrough curve. The values of “q_e_” and “K_TH_” were obtained from the linear plot between $\left(\ln\frac{C_{0}}{C_{t}-1}\right)$ and time (t), as shown in Figure S7 (Supplementary Materials).

Different constants and variables were obtained from the Yoon–Nelson model at various flow rates, as displayed in Table 6, which suggests that the values of KTH were enhanced from 27.19 × 10^−2^ to 43.53 × 10^−2^. Meanwhile, the values of τ (min) declined from 253.18 to 82.80 as the flow rates were increased. The reason is that the MNPs/rGO/PMMA composite in the column saturated more quickly to attain equilibrium as the flow rate increased. The values of R^2^ achieved were 0.998, 0.997 and 0.982 for the three flow rates, respectively. This indicated that the Yoon–Nelson model fitted the experimental adsorption data well, which is consistent with results previously published in the literature [56].

## 6. Adsorption Mechanism

The adsorption system in the current investigation is the expected interaction between hydrogen chromate (HCrO_4_^−^) and functional groups of the adsorbent material. The main factor involved in the mechanism of Cr(VI) adsorption is pH. The most extreme adsorption of Cr(VI) was found to be at pH 2. At this pH, Cr_2_O_7_^2−^ was changed to HCrO_4_^−^ species, as shown in the following reaction [46]:

$${\mathrm{Cr}}_{2}O_{7}^{2-}+{\;H}_{2}O\;\to 2{\mathrm{HCrO}}_{4}^{-}$$

**Scheme 4:**
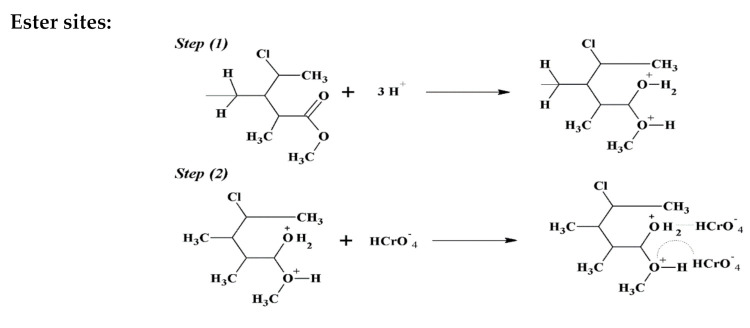

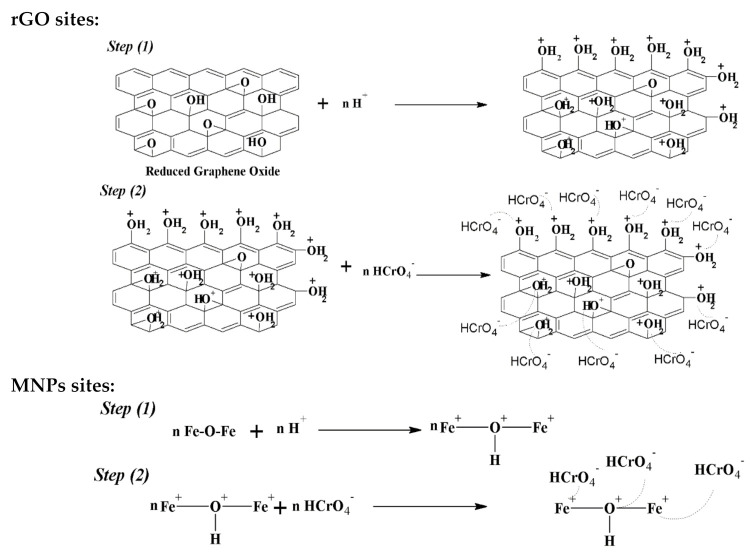
Structure of adsorption mechanism.

The adsorptive capacity of the magnetic composite was due to the active sites present (organic and inorganic phases), as well as the new sites yielded from the interaction between these phases. The adsorption of HCrO_4_^−^ on the active sites of organic and inorganic phases involves an electrostatic interaction. The oxygen atom on the surface of the magnetic composite becomes protonated to a high degree at pH 2, which brings a substantial electrostatic fascination among HCrO_4_^−^ and positively charges on the adsorbent. At this state, hazardous Cr(VI) in tannery waste gets adsorbed onto the surface of MNPs via electrostatic attraction, as shown in Scheme 4.

## 7. Physicochemical Study of Tannery Wastewater

Tannery wastewater was collected from industry and a physicochemical study was carried out before adsorption treatment, as given in Section 3.2. Various physicochemical parameters were analyzed, including pH, COD, suspended solids, Cr concentration and BOD, as presented in Table 8. In the case of tannery wastewater, after the adsorption treatment, both in batch and column modes, the concentration of Cr, level of COD and BOD were reduced to 3.51 and 2.42, 110 and 99, 120 and 109 mg/L, respectively, whereas no suspended solids were found after adsorption, as they were removed through filtration before treatment. These findings conclude that all the parameters are well below the permissible range for the Cr(VI) in wastewater, indicating the effective role of this study in processing and cleaning tannery wastewater.

To the best of our knowledge, the application of MNPs/rGO/PMMA composite materials for the adsorption and recovery of Cr(VI) from real tannery wastewater with a concentration of Cr(VI) as high as 1640 mg/L through both batch and column mode adsorption has not been reported in earlier literature. This material offers multiple functionalities for adsorption of Cr(VI) through a diverse mechanism; hence, it provides higher adsorption potential than any other conventional material, which is explained in detail in Section 6. 

Owing to the hazardous nature of Cr(VI) in water bodies, the removal of Cr(VI) from wastewater streams has been extensively studied in the literature. In this regard, Table 9 presents a comparison of the adsorption efficiency of current adsorbents and various types of other materials reported in the literature, which concludes that the MNPs/rGO/PMMA composite offers superior efficiency.

## 8. Conclusions

A well-organized and novel MNPs/rGO/PMMA composite was successfully fabricated and applied for the adsorptive removal and recovery of Cr(VI) from tannery wastewater. The synthesized composite was analyzed in detail by the XRD, FTIR and SEM techniques. The FTIR investigation declared the successful synthesis of the rGO, MNPs/rGO, PMMA and MNPs/rGO/PMMA composite, while the SEM analysis confirmed the rougher surface morphology of the MNPs/rGO/PMMA composite. In batch adsorption, maximum adsorption of Cr(VI) (99.32 ± 2%) was attained under the optimum conditions of pH 2, sample volume of 10 mL, adsorbent amount of 150 mg and shacking time of 30 min. The kinetic investigations showed that the adsorption followed a pseudo-1st order kinetic model. The experimental data of adsorption followed the Langmuir model. Cr(VI) particles were effectively desorbed from the adsorbent using 30 mL of 2 M NaOH solution, while the adsorbent remained stable for five consecutive reuses. In the case of continuous column mode adsorption, the breakthrough time decreased with the increase in the feed flow rate as well as that of the mass of the composite adsorbent. According to the Thomas model, the Cr adsorption capacity of the composite with a column bed height of 2.7 cm and feed flow rate of 1 mL/min was found to be 135 mg/g. This study provides useful evidence for the remediation of hazardous Cr(VI) from tannery wastewater via a cost-effective and mechanically feasible strategy on a large scale.

## Figures and Tables

**Table 1:** Kinetics parameters, for adsorption of Cr(VI) on MNPs/rGO/PMMA composite; pH 2; contact time, 30 min; adsorbent amount, 150 mg.

Experimental q_e_ (mg·g^−1^)	Pseudo-1st Order			Pseudo-2nd Order		
	K_1_ min^−1^	q_e_ (mg·g^−1^)	R^2^	K_2_ (g.mg^−1^·min^−1^)	q_e_ (mg·g^−1^)	R^2^
109.33	0.151	23.93	0.86	0.362	166.66	0.98

**Table 2:** Adsorption isotherm models, for adsorption Cr(VI) on MNPs/rGO/PMMA composite.

Model Equation	R^2^	Kf	1/n	K_b_	q_m_ (mg·g^−1^)	R_L_
Langmuir Isotherm	0.991	---	---	4.021	240.96	0.199
Freundlich Isotherm	0.972	6.85	0.351	---	---	

**Table 3:** Thermodynamic parameters for the adsorption of Cr(VI) on (MNPs/rGO/PMMA).

Temperature K	ΔG^o^ KJ.mole^−1^	ΔH^o^ KJ.mole^−1^	ΔS^o^ KJ.mole^−1^
293	−14.4944	46.0074	0.1875
303	−15.4132		
313	−16.5329		
323	−17.4261		
333	−18.3889		
343	−19.4415		
353	−20.8456		
363	−22.5964		
373	−24.0239		

**Table 4:** Influence of various alkaline solutions, used for desorption of Cr(VI) from NPs/rGO/PMMA.

Basic Solution	Concentration of Eluent mol·L^−1^	%, Recovery of Cr(VI)
NaOH	1	70 ± 1
NaOH	2	95 ± 1
NH_4_OH	1	65 ± 2
NH_4_OH	2	78 ± 1

**Table 5:** Effect of interfering ions on the adsorption of Cr-VI ions over the MNPs/rGO/PMMA composite.

Ions	Concentration (mg/L)	% Adsorption of Cr(VI)
Cu^2+^	10	96 ± 4
Mn^2+^	10	98 ± 3
Zn^2+^	100	93 ± 1
Cd^2+^	100	97 ± 1
Fe^3+^	100	96 ± 1
Ni^2+^	10	94 ± 1
Ca^2+^	500	98 ± 1
Mg^2+^	500	98 ± 1
CO_3_^2−^	500	96 ± 1
F^−^	1000	99 ± 1
SO_4_^2−^	500	98 ± 1

**Table 6:** Parameters for fixed-bed adsorption of Cr(VI) on MNPs/rGO/PMMA at breakthrough point.

Parameters		Adsorbent Capacity (mg/g)	AER (g/L)
Adsorbent Mass (g)	Flow Rate (mL/min)		
1	2	125.16	20.00
2	2	134.77	17.24
3	2	152.13	14.56
**Flow rate (mL/min)**	**Adsorbent masses (g)**		
1	3	120.06	11.02
2	3	111.98	10.10
3	3	75.01	9.80

**Table 7:** Adsorption parameters calculated from the Thomas and Yoon–Nelson models.

Parameters				Thomas Model			Yoon–Nelson Model		
C_0_ (mg/L)	Rate Flow (mL/min)	Bed Height (cm^−1^)	pH	K_TH_ (L/min gm)	q (mg/g)	R^2^	K_YN_ (min^−1^)	τ (min)	R^2^
1640	1	2.7	2	11.58 × 10^−2^	135.31	0.999	27.19 × 10^−2^	135.31	0.998
1640	2	2.7	2	9.14 × 10^−2^	126.51	0.993	22.03 × 10^−2^	126.51	0.997
1640	3	2.7	2	8.82 × 10^−2^	111.48	0.972	43.53 × 10^−2^	111.48	0.982

**Table 8:** Physicochemical characteristics of tannery wastewater before and after adsorption treatment.

Parameters	Values		
	Before Treatment	After Batch Mode Adsorption	After Column Adsorption
Cr concentration	1640 mg/L	3.51 mg/L	3.42 mg/L
pH	3.17	Variable	Variable
Chemical oxygen demand (COD)	1130 mg/L	110 mg/L	99 mg/L
Biological oxygen demand (BOD)	396 mg/L	120 mg/L	109 mg/L
Suspended solids (SS)	960 mg/L	0.00 mg/L	0.00 mg/L

**Table 9:** Comparison of Cr(VI) adsorption capacities of various adsorbents.

Adsorbent	Adsorption Capacity	pH	Adsorption Process	Wastewater	Ref.
Bagasse fly ash	29.07 mg/g	2–3	Batch	Synthetic	[61]
Fe_3_O_4_/rGO	98.1%	1	Batch	-	[41]
Non-cross-linked chitosan	80 mg/g	5	Batch	Synthetic	[62]
Polymeric based surfactant-chitosan	180 mg/g	5.3	Batch	Synthetic	[63]
Sawdust	1.74 mg/g	3	Batch	Synthetic	[64]
SWCNTs	96.9 mg/g	4	Batch	Synthetic	[65]
MNPs/rGO/PMMA	109.3/135.3 mg/g	3	Batch/column	Wastewater	Current work

## Data Availability

Not applicable.

## Supplementary Materials

The following are available online at https://www.mdpi.com/article/10.3390/ma14226923/s1, Figure S1. Pseud 1st order of kinetic plot for the adsorption of Cr(VI) on MNPs/rGO/PMMA composite. Figure S2. Pseudo 2nd order of kinetic plot for the adsorption of Cr(VI) on MNPs/rGO/PMMA composite, Figure S3. Langmuir adsorption isotherm for the adsorption of Cr(VI) on MNPs/rGO/PMMA composite. Figure S4. Freundlich adsorption isotherm for the adsorption of Cr(VI) on MNPs/rGO/PMMA composite. Figure S5. Enthalpy and entropy changes for the adsorption of Cr(VI) on MNPs/rGO/PMMA composite. Figure S6. Thomas model for various flow rates i.e., 1, 2, and 3 mL/min for the adsorption of Cr(VI) on MNPs/rGO/PMMA composite. Figure S7. Yoon-Nelson model at different flow rates i.e., 1, 2, and 3 mL/min for the adsorption of Cr(VI) on MNPs/rGO/PMMA composite.
