# Supplementary material for: Fabrication of MNPs/rGO/PMMA Composite for the Removal of Hazardous Cr(VI) from Tannery Wastewater through Batch and Continuous Mode Adsorption
Source: Materials (Basel). 2021 Nov 16;14(22):6923. doi: 10.3390/ma14226923 (PMC8620348; doi:10.3390/ma14226923)
Supplement: Supplementary file 1 [file materials-14-06923-s001.zip › materials-1437665-supplementary.pdf]

materials

Article

Fabrication of MNPs/rGO/PMMA Composite for the Removal of Hazardous Cr(VI) from Tannery Wastewater through Batch and Continuous Mode Adsorption

Rahman Ullah [1], Waqas Ahmad [1,*], Muhammad Yaseen [1], Mansoor Khan [2], Mehmood Iqbal Khattak [3], Badrul Mohamed Jan [4,*], Rabia Ikram [4,*] and George Kenanakis [5]

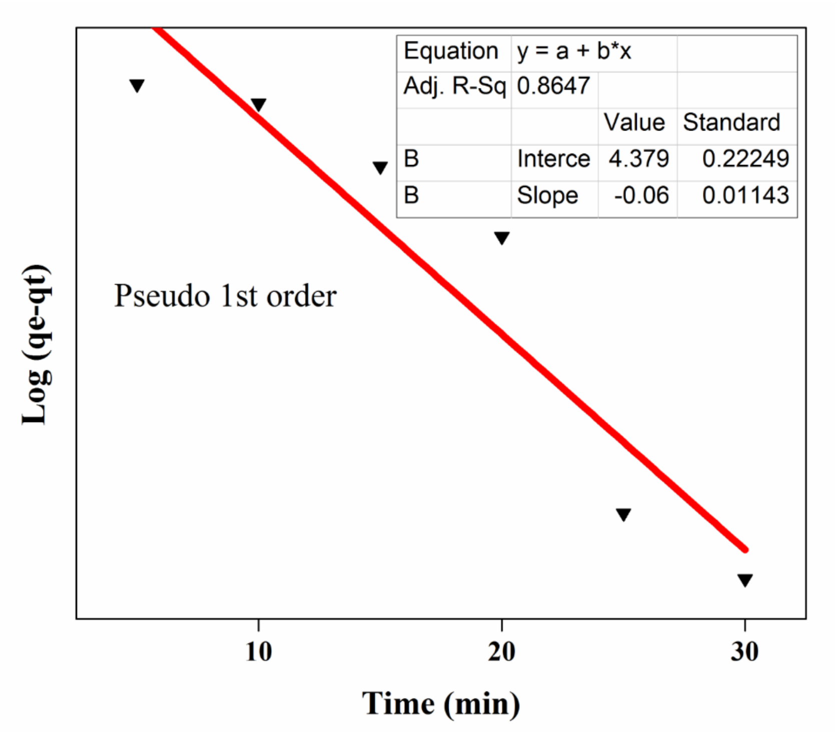

Figure S1. Pseud 1st order of kinetic plot for the adsorption of Cr(VI) on MNPs/rGO/PMMA composite.

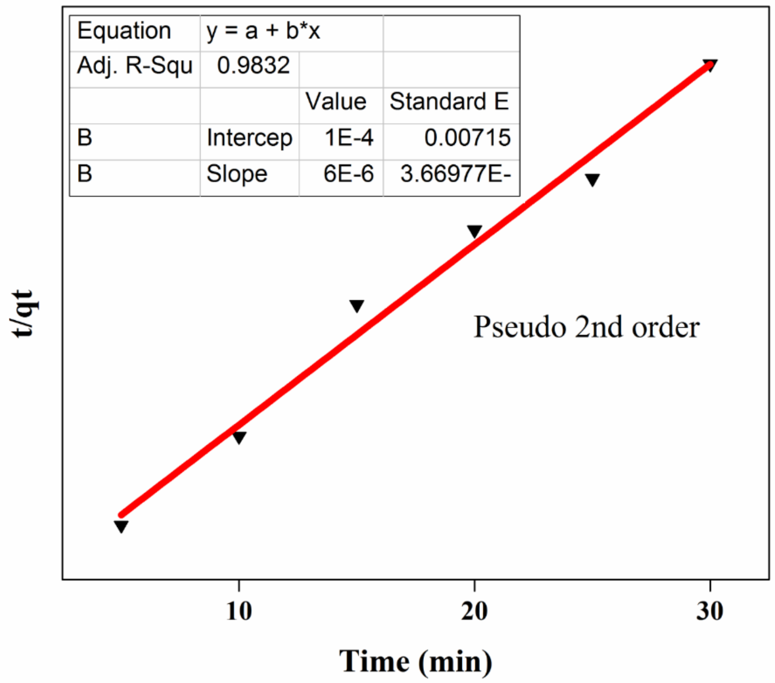

Figure S2. Pseudo 2nd order of kinetic plot for the adsorption of Cr(VI) on MNPs/rGO/PMMA composite.

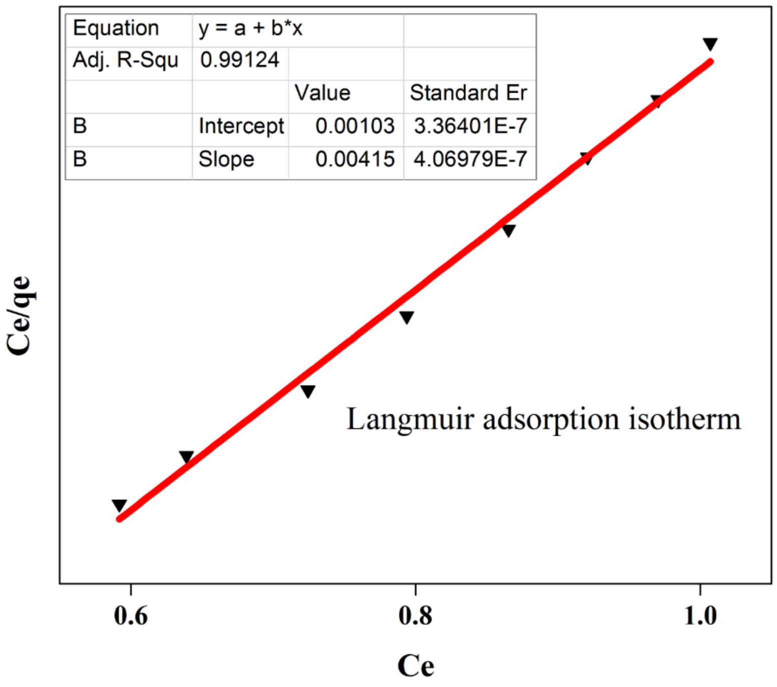

Figure S3. Langmuir adsorption isotherm for the adsorption of Cr(VI) on MNPs/rGO/PMMA composite.

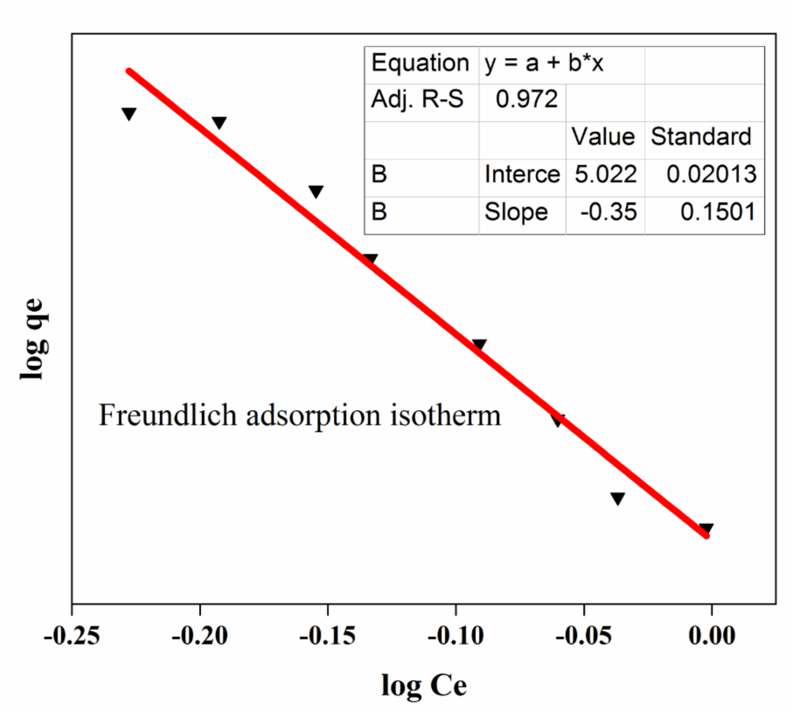

Figure S4. Freundlich adsorption isotherm for the adsorption of Cr(VI) on MNPs/rGO/PMMA composite.

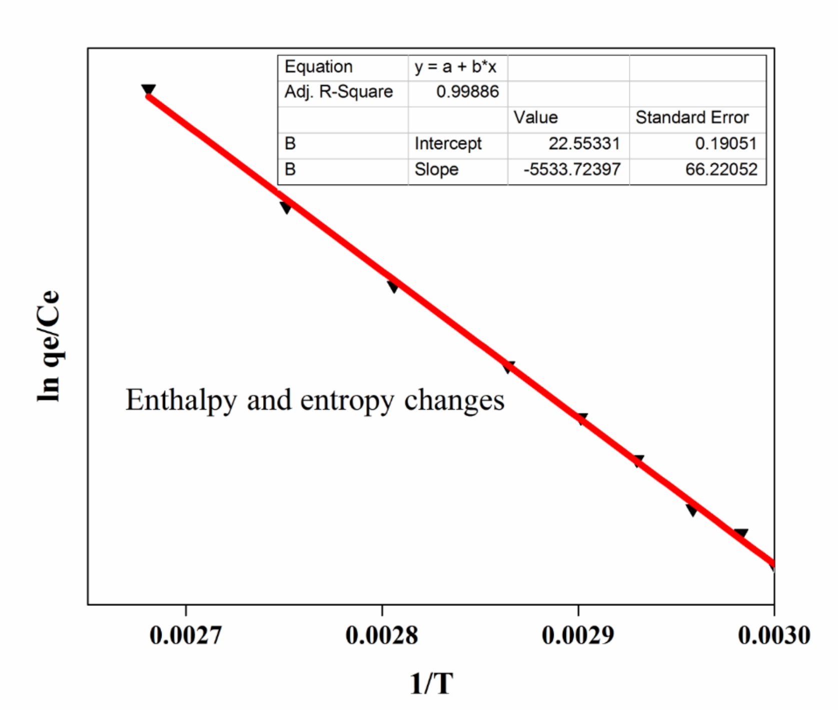

Figure S5. Enthalpy and entropy changes for the adsorption of Cr(VI) on MNPs/rGO/PMMA composite.

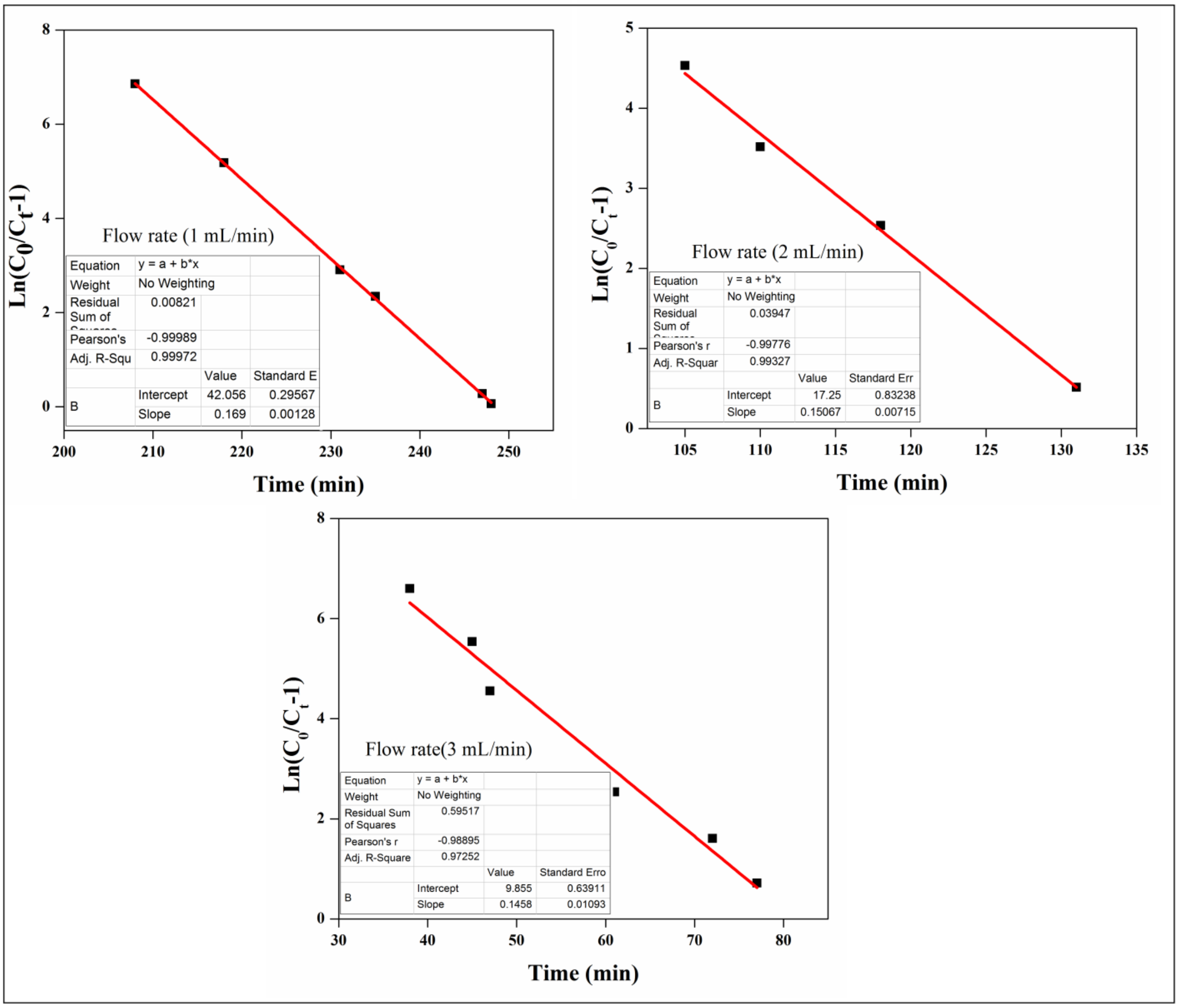

Figure S6. Thomas model for various flow rates i.e. 1, 2, and 3 mL/min for the adsorption of Cr(VI) on MNPs/rGO/PMMA composite.

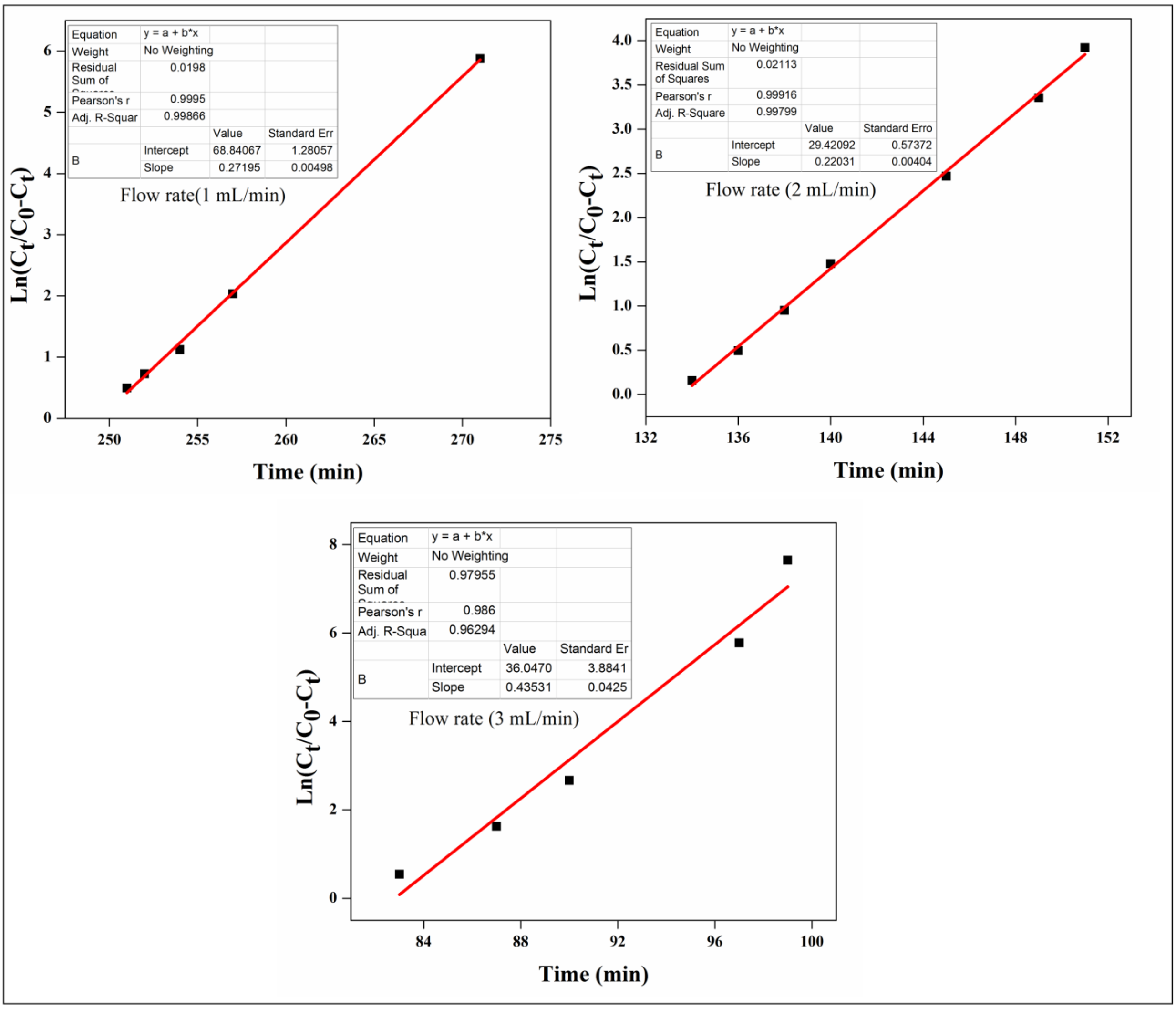

Figure S7. Yoon-Nelson model at different flow rates i.e. 1, 2, and 3 mL/min for the adsorption of Cr(VI) on MNPs/rGO/PMMA composite.
